# Nanoparticles for Stem Cell Therapy Bioengineering in Glioma

**DOI:** 10.3389/fbioe.2020.558375

**Published:** 2020-12-07

**Authors:** Henry Ruiz-Garcia, Keila Alvarado-Estrada, Sunil Krishnan, Alfredo Quinones-Hinojosa, Daniel M. Trifiletti

**Affiliations:** ^1^Department of Radiation Oncology, Mayo Clinic, Jacksonville, FL, United States; ^2^Department of Neurological Surgery, Mayo Clinic, Jacksonville, FL, United States

**Keywords:** biomaterials, nanotechnology, nanoparticles, stem cells, glioma, bioengineering, targeting, surface functionalization

## Abstract

Gliomas are a dismal disease associated with poor survival and high morbidity. Current standard treatments have reached a therapeutic plateau even after combining maximal safe resection, radiation, and chemotherapy. In this setting, stem cells (SCs) have risen as a promising therapeutic armamentarium, given their intrinsic tumor homing as well as their natural or bioengineered antitumor properties. The interplay between stem cells and other therapeutic approaches such as nanoparticles holds the potential to synergize the advantages from the combined therapeutic strategies. Nanoparticles represent a broad spectrum of synthetic and natural biomaterials that have been proven effective in expanding diagnostic and therapeutic efforts, either used alone or in combination with immune, genetic, or cellular therapies. Stem cells have been bioengineered using these biomaterials to enhance their natural properties as well as to act as their vehicle when anticancer nanoparticles need to be delivered into the tumor microenvironment in a very precise manner. Here, we describe the recent developments of this new paradigm in the treatment of malignant gliomas.

## Introduction

Gliomas are a dismal entity, associated with poor survival and high morbidity. The current standard of care has reached a therapeutic plateau even after combining maximal safe resection and chemoradiation (Stupp et al., 2005; Cantrell et al., 2019). In this setting, stem cell therapies have risen as a promising therapeutic approach for gliomas; however, there still exist crucial drawbacks holding its pass to an extensive acceptance in clinical applications. The development of nanomedicine is a parallel phenomenon with potential deep implications in the way stem cells will be introduced into human glioma therapy. Stem cells can be engineered using this nanotechnology in different ways in order to increase our understanding about their biology, improve stem cells antitumor properties, and synergize them with other approaches such as chemotherapy, radiation, thermotherapy, etc. (Kim et al., 2011; Mangraviti et al., 2016; Karlsson et al., 2019; Kozielski et al., 2019; Tian et al., 2020). We aim to provide an overview of the foundations of stem cell therapy and nanoparticles to then explore the potential synergy between these two, through an up-to-date analysis of the benefits of coupling both therapeutic approaches.

## Gliomas

Gliomas are the most common and devastating primary brain tumors, representing approximately 75% of these. According to the World Health Organization (WHO), gliomas are classified in four histological grades (I–IV), being the glioblastomas the corresponding WHO grade IV tumor. Glioblastoma (GBM) is the most common and aggressive among all gliomas, accounting for 57.3% of the tumors in this group, with around 12,500 new cases diagnosed every year only in United States (Cantrell et al., 2019; Ostrom et al., 2019). Glioblastomas present a median overall survival of 15 months and a 5-year survival rate of only 4.6% even after maximal therapy (Cantrell et al., 2019). Furthermore, most of the patients diagnosed with gliomas of lower grade, such as astrocytomas and oligodentrogliomas grade II and III (anaplastic), will eventually progress and perish because of the disease. Overall, these facts are just the translation of the need to develop novel therapeutic approaches able to help extend survival and improve the quality of life of patients with the diagnosis of glioma.

### Limitation of Current Therapies

The current gold standard for the treatment of gliomas includes surgery and chemoradiation. Maximal safe resection is advised in all cases regardless of the WHO grade, given that the overall survival is positively correlated with the extent of resection (EOR) (McGirt et al., 2008, 2009; Chaichana et al., 2014a,b,c; Mahato et al., 2018; Mampre et al., 2018; Marenco-Hillembrand et al., 2020; Suarez-Meade et al., 2020). However, surgery is not curative in any case. Chemotherapy and radiation are required for high-grade gliomas. Anaplastic astrocytomas and oligodendrogliomas (grade III gliomas) will require chemoradiation depending on clinical parameters and tumor molecular characteristics (Caccese et al., 2020). Glioblastoma tumors require postoperative radiotherapy, with concurrent and adjuvant chemotherapy. Unfortunately, despite this multidisciplinary treatment, gliomas will inevitably recur due to their infiltrative nature and high treatment resistance (Cantrell et al., 2019). By the time of surgery, it is estimated that glioma cells have already migrated beyond the macroscopically identifiable tumor, and thereafter, these cells will ultimately seed local recurrence around the surgical cavity (75–80% of cases) and/or non-local recurrence in the reminder 20–25% of cases (Brandes et al., 2009; Chamberlain, 2011; Drumm et al., 2019).

A subset of gliomas cells have been pinpointed as the culprit of this recurrence. The glioma cancer stem cells (CSCs) are a subgroup of malignant cells with the potential of self-renewal, forming tumors that resemble the original pathology, as well as high resistance to current chemotherapeutics and radiation (Singh et al., 2003; Galli et al., 2004; Beier et al., 2007; Li et al., 2009; Cheng L. et al., 2013; Dahan et al., 2014). These cells migrate beyond the macroscopic tumor, infiltrating apparent normal brain parenchyma by the time of surgery and survive even after receiving high-dose radiation and chemotherapy (Li et al., 2009; Lathia et al., 2012). As these cells migrate beyond the tumor bulk to seed further recurrence, a therapeutic strategy able to track these newly developed microscopic glioma foci to deliver antitumor cargoes is of utmost importance. In this setting, the use of neural and mesenchymal stem cells (MSCs) as a therapeutic armamentarium against gliomas represents a potential avenue to achieve this goal and alter the treatment paradigm of this dismal cancer (Pendleton et al., 2013; Li et al., 2014; Smith et al., 2015; Mangraviti et al., 2016).

## Stem Cells as Elements of Therapy for Malignant Glioma

Stem cells have risen as a promising therapeutic option for the treatment of malignant gliomas, as they would be able to migrate and home into glioma tumors, including microscopic tumor foci, which harbor the potential to seed future recurrence (Brown et al., 2003; Nakamura et al., 2004; Kim et al., 2005, 2018; Sonabend et al., 2008; Thu et al., 2009; Yong et al., 2009; van Eekelen et al., 2010; Amano et al., 2011; Choi et al., 2011; Kleinschmidt et al., 2011; Ryu et al., 2011; Altanerova et al., 2012; Jiao et al., 2012; Kosaka et al., 2012; Cheng Y. et al., 2013; Huang et al., 2013, 2014; Lee et al., 2013; Balyasnikova et al., 2014; Li et al., 2014; Mooney et al., 2014b; Bryukhovetskiy et al., 2015; de Melo et al., 2015; Martinez-Quintanilla et al., 2015; Morshed et al., 2015; Park et al., 2015; Cheng S. H. et al., 2016; Kim S. J. et al., 2016; Kim S. M. et al., 2016; Liu et al., 2016; Mangraviti et al., 2016; Muroski et al., 2016; Meca-Cortes et al., 2017; Portnow et al., 2017; Hsu et al., 2018; Lang et al., 2018; Pavon et al., 2018; Tirughana et al., 2018; Zhang et al., 2018; Huang R. Y. et al., 2019; Tanrikulu et al., 2019; Allahverdi et al., 2020; Jabbarpour et al., 2020). Stem cells are relatively easy to grow *in vitro* and can be bioengineered to deliver a wide range of antitumor payloads such as proteins, oncolytic viruses, prodrugs, small interfering RNA (siRNA), and nanoparticles (Brown et al., 2003; Nakamura et al., 2004; Kim et al., 2005, 2018; Sonabend et al., 2008; Thu et al., 2009; Yong et al., 2009; van Eekelen et al., 2010; Amano et al., 2011; Choi et al., 2011; Kleinschmidt et al., 2011; Ryu et al., 2011; Altanerova et al., 2012; Jiao et al., 2012; Kosaka et al., 2012; Cheng Y. et al., 2013; Huang et al., 2013, 2014; Lee et al., 2013; Balyasnikova et al., 2014; Li et al., 2014; Mooney et al., 2014b; Bryukhovetskiy et al., 2015; de Melo et al., 2015; Martinez-Quintanilla et al., 2015; Morshed et al., 2015; Park et al., 2015; Cheng S. H. et al., 2016; Kim S. J. et al., 2016; Kim S. M. et al., 2016; Liu et al., 2016; Mangraviti et al., 2016; Muroski et al., 2016; Meca-Cortes et al., 2017; Portnow et al., 2017; Hsu et al., 2018; Lang et al., 2018; Pavon et al., 2018; Tirughana et al., 2018; Zhang et al., 2018; Huang R. Y. et al., 2019; Tanrikulu et al., 2019; Allahverdi et al., 2020; Jabbarpour et al., 2020).

Stem cells are undifferentiated cells with capacity of self-renewal and differentiation by definition. They can mature along symmetric and asymmetric replication processes. The later type of cell division will result in different hierarchies within stem cell niches, which will now include *progenitor cells*; these are daughter cells retaining the same stem cells properties but with a *de novo* limited differentiation ability (Young et al., 2014).

### Stem Cell Classification

Stem cells can be designated according to their developmental status as *adult*, *fetal*, or *embryonic stem cells*. Their differentiation potential can further define them as *totipotent*, *pluripotent*, or *multipotent stem cells*. *Totipotent stem cells* are only found during the very first days of life just after fecundation and have the capacity to derive into any type of human cells, including placental tissues. Once the embryo has reached the blastocyst stage, cells contained inside the inner cell mass are defined as *pluripotent*, as they can differentiate into any cell of all three germ layers but no placental tissues (Takahashi and Yamanaka, 2006; Qiao et al., 2018; Andres et al., 2019; Klimanskaya, 2019). Eventually, these *pluripotent stem cells* will restrict their differentiation potential to only one of the three germ cell layers and thereafter will be defined as *multipotent stem cells*, which can actually be harvested from most of the organs of the human body (Takahashi and Yamanaka, 2006; Qiao et al., 2018; Andres et al., 2019; Klimanskaya, 2019).

Stem cells used in glioma therapy are usually *multipotent* cells obtained from adult or fetal organs. In particular, neural stem cells (NSCs), mesenchymal stem cells (MSCs), and hematopoietic stem cells (HSCs) are the most common multipotent stem cells used with this purpose (Aboody et al., 2000; Portnow et al., 2017). It is noteworthy, however, that *pluripotent* cells such as the induced-pluripotent stem cells (iPSC) or embryonic pluripotent stem cells have also been described in cell therapy against glioma (Parker Kerrigan et al., 2018; Table 1).

**TABLE 1:** Classification and major features of stem cells reported in glioma therapy.

Stem cell	Defining criteria	Source/Niche	Linage
**Pluripotent stem cells: capacity to differentiate into any cell of all three germ layers**			

Embryonic stem cells (ECSs)	Markers of pluripotency as found in ICM cells: **Transcription factors:** Oct4, Nanog, Rex-1 **Cell surface markers:** SSEA-3, SSE-A4, TRA-1-60, TRA-1-81, alkaline phosphatase	Blastocyst Morula Growth-arrested embryos Somatic cell nuclear transfer Single blastomere	As defined by pluripotency
Induce pluripotent stem cells (iPSCs)	Essentially the same than ECS markers	Reprogrammed adult somatic cells—usually fibroblasts or skin cells.	As defined by pluripotency given than iPSC are functionally equivalent to ECS

**Multipotent stem cells: capacity to differentiate into cells of one of the three germ layers**			

Neural stem cells (NSCs)	**Positive:** GFAP, CD133, CD184, and Nestin, Sox1, Sox2, and Pax6 **Negative:** CD271, CD44, CD24 **Immune profile:** Absent HLA II	*Subependymal zone* (SEZ)—lining the lateral ventricles *Dentate gyrus* of the hippocampus *Obtained from fetal and adult mammals	Neurons Oligodendrocytes Astrocytes Ependymal cells
Mesenchymal stem cells (MSCs)	MSC must comply with ISCT criteria: **Positive (>95% +):** CD105, CD73, CD90 **Negative (<2% –):** CD45, CD34, CD14 or CD11b, CD79α, or CD19, HLA DR Adherence to plastic in standard culture conditions *In vitro* differentiation to osteoblast, adipocytes, and chondroblasts. **Immune profile:** Absent HLA II	***Adult tissues:*** adipose tissue, bone marrow, peripheral blood, dental pulp, ligamentum flavum, synovium, endometrium, sweat glands, and milk **Fetal tissues:** umbilical cord, umbilical cord blood, Wharton jelly, amniotic fluid, chorionic villi, and placenta	Osteoblast Adipocytes Chondroblast *Differentiation into ectodermal and endodermal linages has also been reported
Hematopoietic stem cells (HMSs)	**Negative:** CD45R/B220 (B cells), Gr-1 (granulocytes), Mac-1 (macrophages), Ter-119 (erythrocytes) and CD4/CD8 (lymphocytes)—for phenotypic enrichment. **Positive:** Sca-1, c-Kit, CD150 *They appear as side population in dye exclusion assays due to the high expression of MDR pumps	***Adult tissues:*** bone marrow, peripheral blood **Non-adult tissues:** umbilical cord blood, yolk sac, liver, spleen	Hematopoietic cells

**IMC, inner mass cells; MDR, multidrug resistance.**

### Development of Stem-Cell-Based Glioma Therapy

The use of stem cells in glioma therapy relies on their tumor-homing properties. This property was first described in 2000 by Aboody et al. The group presented a seminal paper describing the glioma tropism of *neural stem cells*. The study reported on the capacity of NSC for engrafting into the glioma bulk when intratumor NSC injections were performed, invading normal parenchyma only when tumor cells migrate far from the tumor mass; with this, they also showed the specific NSC ability to track glioma cancer cells infiltrating along healthy tissue. NSCs were also proven to migrate toward glioma tumor masses when implanted distally to these, through ipsilateral, contralateral, and intraventricular NSC injections (Aboody et al., 2000). These abilities and the possibility of being bioengineered to secrete antiglioma cargoes turned NSC into a promising glioma treatment, able to track and tackle this infiltrative malignant tumor. Importantly, NSC showed to retain their stem cell properties and had been proven non-tumorigenic (Snyder et al., 1992). In 2017, the same group published the first phase I clinical trial where NSC-based antiglioma therapy was proven safe; the proof of concept of NSC tumor homing was also demonstrated by the group (Portnow et al., 2017). Unfortunately, despite the encouraging role of NSC in glioma therapy, limited availability of human NSC (hNSC) as well as ethical concerns regarding its use encouraged researchers to seek alternatives sources of stem cells.

*Mesenchymal stem cells (MSCs)* were first described by Friedenstein more than 50 years ago (Friedenstein et al., 1968, 1970, 1974). He initially isolated MSC from rodent bone marrow and proved that they were able to differentiate into mesenchymal tissue (adipogenic, chondrogenic, and osteogenic differentiation). MSCs ended up being an alternative to the difficult-to-obtain NSC, as they are abundant in several adult and fetal tissues such as bone marrow (BM-MSC) (Friedenstein et al., 1968, 1970, 1974), adipose tissue (A-MSC) (Zuk et al., 2001; Katz et al., 2005; Wagner et al., 2005), umbilical cord (UC-MSC) (Girdlestone et al., 2009), umbilical cord blood, Wharton jelly (Erices et al., 2000; Zeddou et al., 2010), endometrium (Meng et al., 2007), dental pulp (Agha-Hosseini et al., 2010), ligamentum flavum (Chen et al., 2011), etc. (Kassis et al., 2006; Miao et al., 2006; Roubelakis et al., 2007; Poloni et al., 2008; Patki et al., 2010; Ma et al., 2018). MSCs are easy to harvest and isolate even from adult individuals, which would allow for using patient-derived MSC as autografts in glioma patients, thus avoiding ethical dilemmas as well as fears about immune-mediated allograft rejection. In this context where MSCs could be isolated from a variety of tissue sources, cultured following different methodologies, and be defined by using different surface markers, the Mesenchymal and Tissue Stem Cell Committee of the International Society for Cellular Therapy (ISCT) proposed a standard set of minimum criteria defining MSC for both laboratory-based scientific investigations and preclinical studies (Dominici et al., 2006). Thus, every study currently under development should follow these guidelines in order to assure a better cell homogeneity among different laboratories and greater reproducibility.

The first report on the use of MSC in the treatment of gliomas came from Nakamura et al. In 2004, the group demonstrated that MSCs also possessed glioma-homing properties by proving rat-derived BM-MSC homing in a rat glioma model. In 2005, Nakamizo et al. were able to replicate the findings using human BM-MSC on a murine model harboring glioma xenograft derived from commercial human cell lines (U87, U251, and LN229). Furthermore, both groups were able to bioengineer the MSCs to deliver antitumor cargoes. To date, several reports on the use of MSC as key elements for glioma stem cell therapy have been published with exceptional promising results (Table 2; Brown et al., 2003; Nakamura et al., 2004; Kim et al., 2005, 2018; Sonabend et al., 2008; Thu et al., 2009; Yong et al., 2009; van Eekelen et al., 2010; Amano et al., 2011; Choi et al., 2011; Kleinschmidt et al., 2011; Ryu et al., 2011; Altanerova et al., 2012; Jiao et al., 2012; Kosaka et al., 2012; Cheng Y. et al., 2013; Huang et al., 2013, 2014; Lee et al., 2013; Balyasnikova et al., 2014; Li et al., 2014; Mooney et al., 2014b; Bryukhovetskiy et al., 2015; de Melo et al., 2015; Martinez-Quintanilla et al., 2015; Morshed et al., 2015; Park et al., 2015; Cheng S. H. et al., 2016; Kim S. J. et al., 2016; Kim S. M. et al., 2016; Liu et al., 2016; Mangraviti et al., 2016; Muroski et al., 2016; Meca-Cortes et al., 2017; Portnow et al., 2017; Hsu et al., 2018; Lang et al., 2018; Pavon et al., 2018; Tirughana et al., 2018; Zhang et al., 2018; Huang R. Y. et al., 2019; Tanrikulu et al., 2019; Allahverdi et al., 2020; Jabbarpour et al., 2020).

**TABLE 2:** Stem cell therapy in glioma.

Stem cell type	Delivery routes	Applications
**Neural stem cells^*a,b*^**	• Intravascular: vein(Brown et al., 2003) • Intracranial (Mooney et al., 2014b; Morshed et al., 2015) • Intraventricular • Intracerebral (Altanerova et al., 2012; de Melo et al., 2015)	**Prodrug activating enzymes**: CD (Portnow et al., 2017), rCE, and hCE1m6 (Mooney et al., 2014b), HSV-TK (Uhl et al., 2005; Tamura et al., 2020) **Oncolytic viruses**: CRAd-S-pk7 (Morshed et al., 2015) **Cargo proteins**: IL-4 (Benedetti et al., 2000), IL-12 (Ehtesham et al., 2002), PF-4 (Lee et al., 2003), TRAIL (Balyasnikova et al., 2011), PEX (Kim et al., 2005), BMP4 (Liu et al., 2016), TSP-1 (van Eekelen et al., 2010) **Nanoparticles** (Mooney et al., 2014b)
		• Stem cells tracking: FE-Pro (Thu et al., 2009), FTD (Kim S. J. et al., 2016) via MRI, MSN (Cheng S. H. et al., 2016) via SPECT/CT • Payload release: SD (Muroski et al., 2016)**, MSN-Dox (Cheng Y. et al., 2013)
**Mesenchymal stem cells**		
Adipose-derived	• Intranasal (Balyasnikova et al., 2014) • Intracranial (Altanerova et al., 2012; de Melo et al., 2015)	**Prodrug activating enzymes**: yeast CD (Altanerova et al., 2012), HSV-TK (Meca-Cortes et al., 2017)^(c)^, (de Melo et al., 2015) **Oncolytic viruses**: ICOVIR17 (Martinez-Quintanilla et al., 2015) **Cargo proteins**: TRAIL (Balyasnikova et al., 2014; Tanrikulu et al., 2019) **Oligonucleotides**: miR-4731 (Allahverdi et al., 2020)
Bone marrow-derived	• Intratumoral (Kosaka et al., 2012; Lee et al., 2013): alginate microencapsulated (Kleinschmidt et al., 2011) • Intracarotid (Yong et al., 2009)	**Prodrug activating enzymes**: CD (Kosaka et al., 2012), HSV-TK (Amano et al., 2011) **Oncolytic viruses**: Delta24-RGD (Yong et al., 2009), CRAd (Sonabend et al., 2008)*** Cargo Proteins: IL2 (Nakamura et al., 2004), INF-B (Park et al., 2015), TRAIL (Choi et al., 2011), BMP4 (Li et al., 2014; Mangraviti et al., 2016) **Oligonucleotides**: miR-124 (Lang et al., 2018; Lee et al., 2013) and miR-145 (Lee et al., 2013), miRNA-584-5p (Kim et al., 2018) **Nanoparticles**:
		• Gene therapy: MTN (TRAIL) (Huang R. Y. et al., 2019) • Intrinsic MSC modification: IO MNP (improve MSC homing) (Huang et al., 2014) • Stem cell tracking: MNP (Huang et al., 2013), FTD (Kim S. J. et al., 2016) via MRI. NIR675 (Kim S. M. et al., 2016) via near-infrared imaging
Human placenta-derived	• Intratumoral (Lee et al., 2013)	**Cargo proteins**: NK4 (Jabbarpour et al., 2020) **Oligonucleotides**: miR-124 and miR-145 (Lee et al., 2013) **Nanoparticles**
		• Stem cell tracking: PEG-SPIO (Hsu et al., 2018) via MRI
Umbilical cord-derived	• Intratumoral (Lee et al., 2013) • Intravascular: tail vein (Pavon et al., 2018)	**Cargo proteins**: IL12 (Ryu et al., 2011) **Oligonucleotides**: miR-124 and miR-145 (Lee et al., 2013) **Nanoparticles** • Stem cell tracking: MION-Rh (Pavon et al., 2018)*
Amniotic membrane-derived	• Intratumoral (Jiao et al., 2012)	**Direct antiglioma properties**: increased apoptosis (Jiao et al., 2012)
Hematopoietic progenitor cells	• *In vitro* (Bryukhovetskiy et al., 2015)	Migration in an *in vitro* model (Bryukhovetskiy et al., 2015)

CD, cytosine deaminase; rCE, rabbit carboxylesterase; hCE1m6, modified human carboxylesterase; MSN-Dox, doxorubicin loaded-mesoporous silica nanoparticles; FE-Pro, ferumoxide–protamine sulfate complex; FTD, FerraTrack Direct; NIR675, NEO-LIVE, Magnoxide 675 nanoparticles; MSN, mesoporous silica nanoparticles; MTN, magnetic ternary nanohybrid; IO MNP, iron-based magnetic nanoparticles; MNP, mesoporous nanoparticles; NK4, hepatocyte growth factor antagonist; PEG-SPIO, polyethylene glycol-superparamagnetic iron oxide; PF-4, platelet factor 4.

*MSC were found to promote tumor growth.

**2u magnetic disks.

***Only specified as human MSC.

*^*a*^GMP production and scale-up of these cells have been performed (Tirughana et al., 2018).*

*^*b*^First-in human studies have been performed assessing safety of intracranial injection (Portnow et al., 2017).*

*^*c*^Modified MSC produced by CRISPR/Cas9.*

On the other hand, not all are in agreement, as there has been a risen controversy in which some authors have described that MSCs could eventually support glioma tumor growth. Different types of MSCs such as BM-MSC, A-MSC, and UC-MSC have been associated with these proglioma effects through increased proliferation, cancer cells migration, angiogenesis, transition to epithelial–mesenchymal phenotype, and decreased glioma apoptosis (Iser et al., 2016; Ridge et al., 2017); however, this adverse phenotype would vary on a differential basis depending on specific glioma tumors (Breznik et al., 2017). In this same line, brain tumor-derived MSC (BT-MSC) have also been described in mouse- and human-derived glioma tumors supporting glioma microenvironment (Behnan et al., 2014; Guo et al., 2014; Svensson et al., 2017; Yi et al., 2018). In support to these findings, Shahar et al. showed that higher percentages of human BT-MSC directly correlate with worse patient prognosis (Shahar et al., 2017). Overall, these data would suggest that stem cell therapy should be carefully selected in future translational efforts.

In order to improve different aspects of stem cell therapies against glioma, different approaches have been studied. The use of nanoparticles for stem cell bioengineering is one of these potential approaches and will be discussed in the following section.

## Nanoparticles as Elements of Therapy for Malignant Glioma

Nanoparticles (NPs) are defined as nanomaterials sized between 1 and 100 nm in at least one of their external dimensions, which confer them a high surface/volume ratio (European Commission, 2011). Due to this small size, they present significantly different properties when compared to conventional materials of non-nanometric scale. The optical, magnetic, electronic, and biological properties of these nanomaterials can be tuned by size, shape, surface modifications (functionalization), or even by combining them with different materials in order to create new heterostructured nanoparticles (Thimsen et al., 2014; Lee et al., 2017).

In Nano-oncology, nanoparticles represent an important diagnostic and therapeutic tool, as they can be designed to interact with most biological system with great precision and specificity. This is possible due to their particular physicochemical characteristics and the possibility of making them able to target a specific tissue, specific cell types, or a specific cellular compartment (targeted functionalization) (Portney and Ozkan, 2006). The potential benefits of these nanomaterials in medicine have led some of them to obtain Food and Drug Administration (FDA) approval to be investigated under several clinical protocols (Table 3).

**TABLE 3:** Advances in the uses of nanoparticles in glioma therapy and diagnosis.

Use	Experimental setting and nanoparticle type
**Nanocarrier:** Drug bioavailability/therapeutic efficacy enhancer • Usually loaded with drugs such as doxorubicin or biological agents such as siRNA (Yu et al., 2017) • Usually functionalized with ligands of common GBM membrane proteins	Preclinical: • *In vivo*: PLGA-NP (Sousa et al., 2019; Ye et al., 2019; Caban-Toktas et al., 2020; Chung et al., 2020), RGD-NP (Ullah et al., 2020), oleic acid NP (Wang H. et al., 2020) • *In vitro* only: PLGA-NP (Luque-Michel et al., 2019; Ferreira et al., 2020; Roberts et al., 2020), ethyl arachidate (TPLN) (Alves et al., 2020), FONP (Daniel et al., 2019), pSiNPs
**Standalone therapy**	Preclinical • *In vitro*: Selenium NP (Xu et al., 2020), MNP (Shen et al., 2017) • *In vivo*: MP (Cheng Y. et al., 2016; Kim et al., 2010)
**Drug sensitizer**	Temozolomide: direct attenuation on EGFR and MET signaling, through delivered miRNAs (Meng et al., 2020)
**Imaging technologies enhancer**	Fluorescence: USPION (Denora et al., 2019)
**Magnetic hyperthermia**	*Clinical* • Phase I and II: SPION (Maier-Hauff et al., 2007; Wegscheid et al., 2014)
	*Preclinical* • *In vivo*: SPIONa (Rego et al., 2019, 2020; Shi et al., 2019)
**Sonodynamic therapy**	Preclinical • *In vivo*: (Liang et al., 2020)
**Radiotherapy enhancer**	Charged particles
	• Proton (Martinez-Rovira et al., 2020) • Helium • Carbon • Oxygen
	Photon therapy (X-rays) • *In vitro* only: AuNP (Kunoh et al., 2019) • *In vivo*: FA-AuNC (Kefayat et al., 2019), PEGylated-AgNP (Zhao et al., 2019), PEGylated-liposome (Hua et al., 2018), AGuIX (Dufort et al., 2019)*
**Photodynamic therapy enhancer**	Preclinical • *In vivo*: 5-ALA (Wang X. et al., 2020), AuNS (Zhu et al., 2020)^*a*^, ICG (ZhuGe et al., 2019)
**Immunotherapy enhancer**	Functionalization with anti-PDL1 (Ruan et al., 2019; Zhang et al., 2019)

SPION, superparamagnetic iron oxide nanoparticles; USPION, ultrasmall SPION; PLGA, poly-lactide-co-glycolic acid; PEG, polyethylene glycol; RGD, arginyl-glycyl-aspartic tripeptide; TPLN, FONP, fluorescent organic nanoparticles; pSiNPs, porous silicon nanoparticles; terpolymer-lipid nanoparticles; MP, permalloy magnetic particles; FA-AuNC, folic acid gold nanoclusters; AuNP, gold nanoparticles, AgNP, silver nanoparticles; AGuIX, gadolinium-based nanoparticle; 5-ALA, 5-aminolevulinic acid; AuNS, gold nanospheres; ICG, indocyanine green; HSPA5, heat shock protein A5r4t. *Theranostic NP: Possesses diagnostic and therapeutic functions. ^*a*^Improve CT/MRI imaging and also works as radiosensitizer by AuNS properties and loading an HSPA inhibitor.

### Targeted Functionalization

In order to achieve a targeted distribution at a cellular or even intracellular level, NPs can be functionalized via active targeting. Active targeting is achieved by different methods; a method called *ligand targeting* works by coating the nanoparticles’ surface with one or more ligands such as transferrin, epidermal growth factor (EGF), folic acid, arginyl-glycyl-aspartic tripeptide (RGD) peptide, hyaluronic acid, antibodies, and others (Ruiz-Garcia et al., 2020). These ligands allow NPs to bind specific “receptors” differentially expressed only in certain cancerous blood vessels and/or tumor cells, thus leading to a precise cellular internalization (Maier-Hauff et al., 2007; Kim et al., 2010; Wegscheid et al., 2014; Cheng Y. et al., 2016; Shen et al., 2017; Yu et al., 2017, 2019; Hua et al., 2018; Daniel et al., 2019; Denora et al., 2019; Dufort et al., 2019; Kefayat et al., 2019; Kunoh et al., 2019; Luque-Michel et al., 2019; Rego et al., 2019, 2020; Ruan et al., 2019; Shi et al., 2019; Sousa et al., 2019; Ye et al., 2019; Zhang et al., 2019; Zhao et al., 2019; ZhuGe et al., 2019; Alves et al., 2020; Caban-Toktas et al., 2020; Chung et al., 2020; Ferreira et al., 2020; Kazmi et al., 2020; Liang et al., 2020; Martinez-Rovira et al., 2020; Meng et al., 2020; Roberts et al., 2020; Ullah et al., 2020; Wang H. et al., 2020; Wang X. et al., 2020; Xu et al., 2020; Zhu et al., 2020; see Table 3 for examples of *ligand targeting* in glioma research).

Another active targeting method to increase functional specificity of NPs that are used as gene delivery systems is the *transcriptional targeting*, which can occur at a transcriptional or posttranscriptional level (Golombek et al., 2018). Here, the delivered gene includes a tumor-specific promoter (highly functional only in cancer cells), which will secure a well-localized expression of the transgene, limited to occur only inside the cancer cells of interest. Posttranscriptional regulations of the product encoded by the exogenously delivered gene are achieved by controlling RNA splicing, RNA stability, and initiation of the RNA translation once it is present in the cancer cell (Maier-Hauff et al., 2007; Kim et al., 2010; Wegscheid et al., 2014; Cheng Y. et al., 2016; Shen et al., 2017; Yu et al., 2017; Hua et al., 2018; Daniel et al., 2019; Denora et al., 2019; Dufort et al., 2019; Kunoh et al., 2019; Luque-Michel et al., 2019; Ruan et al., 2019; Rego et al., 2019, 2020; Shi et al., 2019; Sousa et al., 2019; Ye et al., 2019; Zhang et al., 2019; Zhao et al., 2019; ZhuGe et al., 2019; Alves et al., 2020; Caban-Toktas et al., 2020; Chung et al., 2020; Ferreira et al., 2020; Kazmi et al., 2020; Liang et al., 2020; Martinez-Rovira et al., 2020; Meng et al., 2020; Roberts et al., 2020; Ullah et al., 2020; Wang H. et al., 2020; Wang X. et al., 2020; Xu et al., 2020; Zhu et al., 2020).

To date, several nanoparticles have shown to be effective in improving different aspects of traditional and novel cancer therapeutic approaches, to the point that several nanocarriers and nanoradiotherapy enhancers are being studied in phase II and III clinical trials (Maier-Hauff et al., 2007; Kim et al., 2010; Wegscheid et al., 2014; Cheng Y. et al., 2016; Shen et al., 2017; Yu et al., 2017; Hua et al., 2018; Daniel et al., 2019; Denora et al., 2019; Dufort et al., 2019; Kunoh et al., 2019; Luque-Michel et al., 2019; Rego et al., 2019, 2020; Ruan et al., 2019; Shi et al., 2019; Sousa et al., 2019; Ye et al., 2019; Zhang et al., 2019; Zhao et al., 2019; ZhuGe et al., 2019; Alves et al., 2020; Caban-Toktas et al., 2020; Chung et al., 2020; Ferreira et al., 2020; Kazmi et al., 2020; Liang et al., 2020; Martinez-Rovira et al., 2020; Meng et al., 2020; Roberts et al., 2020; Ullah et al., 2020; Wang H. et al., 2020; Wang X. et al., 2020; Xu et al., 2020; Zhu et al., 2020). In the next section, we will briefly review the role of nanoparticles as a standalone therapeutic approach for glioma tumors (Table 3), and then, we will review in detail the role of nanoparticles as a tool to further improve stem cell therapy (Table 4).

**TABLE 4:** Rational for a combinatorial approach using nanoparticles and stem cell therapy in malignant glioma.

Rational for nanoparticle-based stem cell therapy in malignant glioma	
Drawbacks of using nanoparticles alone	• Big nanoparticles could be engulfed by the phagocytic mononuclear system (macrophages/lymphocytes) depending on their size • Necrotic central core and histological heterogeneity predispose to uneven intratumor biodistribution of nanoparticles • Infiltrative cells leaving the tumor bulk are unlikely to be tracked by nanoparticles
Potential advantages of using combine therapy	• Stem cells can transport bigger nanoparticles, increasing nanoparticle loading capacity • Stem cells could transport big nanoparticles through the BBB • Stem cells could better deliver therapeutic nanoparticles into the hypoxic central glioma core where treatment-resistant CSC locate • Stem cells could track and deliver their cargo to CSC leaving the tumor bulk. These CSC have been pinpointed as culprits of future tumor recurrence

**BBB, blood brain barrier; CSC, cancer stem cells.**

### Classification

Nanoparticles can naturally occur in the environment mediated by biological or geological processes (Sharma et al., 2015), or as incidental by-product of human activities such as smelting or other processes involving the generation of metal fumes (Gonzalez-Pech et al., 2019). In addition, nanoparticles can be artificially synthetized and engineered (Kus et al., 2018). Given the wide variety of existing NPs, classification criteria are also abundant. We present the classification of NPs according to their origin and structure, as they will help understand the terminology used to describe NPs used in cancer research.

#### Classification of Nanoparticles Based on Its Origin

##### Organic

Organic nanoparticles are based on natural compounds such as lipids, glycosides, peptides and others, as well as synthetic organic molecules (Romero and Moya, 2012; Tzeng et al., 2016; Kus et al., 2018; Karlsson et al., 2019; Kozielski et al., 2019; Tian et al., 2020). These organic elements can arrange themselves in three-dimensional (3D) structures (Euliss et al., 2006), which is one of the main characteristics that differentiate organic from inorganic nanoparticles, as inorganic NPs do not form these 3D structures in any case (Romero and Moya, 2012). Furthermore, due to the weak interactions that hold many organic NPs together, they present a dynamic nature that allows, for example, for fusion and generation of larger structures depending on external conditions (Romero and Moya, 2012).

**FIGURE 1:**
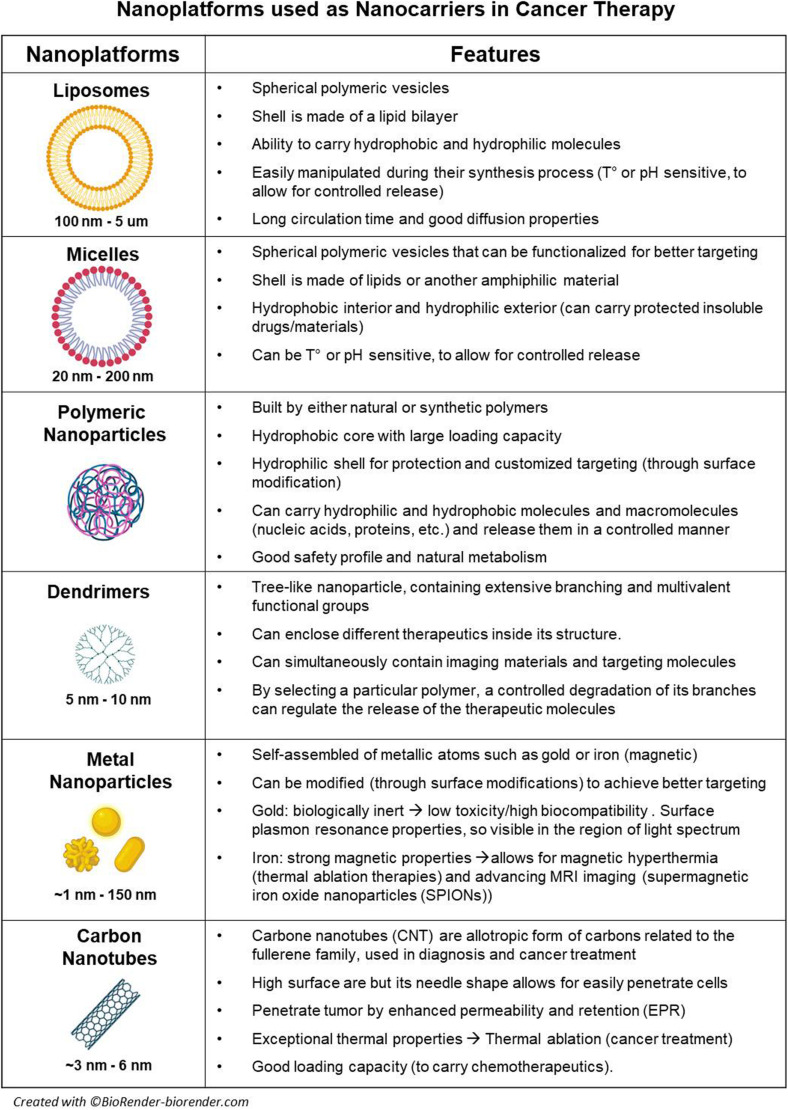
Nanoplatforms used as nanocarriers in cancer therapy.

Organic nanoparticles display highly desirable characteristics in the biomedical field (Hussein Kamareddine et al., 2019). They have a dynamic nature and are able to respond to environmental variations in temperature, pH, and UV radiation (Jagannathan et al., 2006; Affram et al., 2017; Hussein Kamareddine et al., 2019). Furthermore, they can easily cross biological barriers and are considered less toxic due to its biodegradability; therefore, they are ideal as drug or gene delivery systems (Jagannathan et al., 2006; Hussein Kamareddine et al., 2019). Liposomes, vesicles, micelles, polymeric NPs, and dendrimers are all among the most common organic nanoparticles (for specific characteristic and applications, see Figure 1); however, among all of them, polymeric NPs are probably the most relevant in cancer research.

Polymeric NPs, also known as polymeric nanospheres, are commonly defined as solid polymer particles with matrix type structure, where a cargo can be embedded within the polymer matrix or included in the surface (Reis et al., 2006). Based on its origin, polymeric NPs can be classified as natural or synthetic. The first group contains NPs such as chitosan, which is a widely available natural cationic carbohydrate polymer approved by the Food and Drug Administration (FDA) and the European Medicines Agency (EMA) for drug and gene delivery and tissue engineering in humans (Lara-Velazquez et al., 2020). The second group, or synthetic polymer-based nanoparticles (SP-NPs), is the most relevant in medicine, as they can be easily synthesized and their properties can be tailored according to specific needs.

SP-NPs are prepared using synthetic polymers (Romero and Moya, 2012), which can be classified in *polyesters* such as poly(glycolic acid) (PGA), poly(lactic acid) (PLA), poly(caprolactone) (PCL), and poly(lactic-co-glycolic acid) or (PLGA); *polyalkyl alcohols* such as polyvinyl alcohol or PVA; and *polyethers* such as poly(ethylene glycol) (PEG) and poly(propylene glycol) (PPG) (Ranganathan et al., 2018). Currently, there are around 15 FDA-approved nanomedicines based on SP-NPs, 6 of them are used in cancer therapy (Bobo et al., 2016; Farjadian et al., 2019). Up to date, glioma research based on SP-NPs has been mainly focused on the development of more effective delivery systems, able to cross the blood brain barrier and specifically target the cancer cells (Ambruosi et al., 2006; Hua et al., 2011; Jiang et al., 2011; Guo et al., 2013; Bishop et al., 2016; Tzeng et al., 2016; Karlsson et al., 2019; Kozielski et al., 2019). This includes the generation of hybrid systems using booth synthetic polymers and natural compounds (Agrawal et al., 2015; Cook et al., 2015; Alex et al., 2016; Wang et al., 2017; Qi et al., 2020), as well as smart nanoparticles able to react according to the surrounded conditions or to specific stimulus (Soppimath et al., 2005; McNeeley et al., 2009; An et al., 2015; Mangraviti et al., 2015; Gao et al., 2016; Ye et al., 2019).

##### Inorganic

Inorganic nanoparticles present unique physicochemical properties (optical, magnetic, etc.), inertness, high stability, and easy functionalization, which give them different advantages when compared to organic NPs. Due to their cellular internalization ability and low immunogenic response, these nanoparticles were initially used as drug and gene delivery systems (Xu et al., 2006; Evans et al., 2019). Different types of elements and inorganic compounds based on metals [metal NP (mNP)], metalloids, or non-metals such as gold, silver, iron, magnesium, silicon, and others are differentially arranged and/or combined in order to display specific properties. Thus, there are some particular groups of NPs such as *magnetic nanoparticles*, which are usually based in a core of iron oxide mNP with a large magnetic momentum under an external magnetic field, which allow its use as MRI contrast enhancer and thermotherapy agents (Maier-Hauff et al., 2007; van Landeghem et al., 2009; Wegscheid et al., 2014). *Plasmonic nanoparticles* refer to mNPs such as gold (Au) or silver (Ag) NPs presenting with surface plasmon resonance (SPR), meaning that NP free electrons can be excited by electromagnetic fields (UV or infrared light) and resonate, creating the possibility to sense these changes (biosensors), produce heat (photothermal ablation/therapy), or create technologies such as surface-enhanced Raman spectroscopy (SERS) (Kaur et al., 2016; Chen et al., 2018; Liu et al., 2018). *Quantum dots (Qdots)* are another group of inorganic NPs, usually smaller than 50 nm; these semiconductor NPs efficiently produce bioluminescence once excited by UV light, which has led them to be used in single cell and *in vivo* imaging (Xu et al., 2006; Figure 1).

##### Carbon Based

These nanoparticles are predominantly composed by carbon, and their discovery revolutionized diverse scientific fields (Cha et al., 2013; Patel et al., 2019). Carbon-based nanomaterials have outstanding properties like high mechanical strength, thermoelectrical conductivity, and flexibility (Cha et al., 2013). These nanoparticles include fullerenes (carbon nanotubes), graphene, and nanodiamonds. Their broad range of properties makes these materials ideal imaging agents for tumor diagnosis (Patel et al., 2019; Figure 1).

#### Classification of Nanoparticles Base on Their Structure

##### Single Nanoparticles

Single nanoparticles are made of a single element such as gold, silver, copper, among others, and due to their homogeneity and electrochromic properties, they are widely used in electro-optical applications, energy conversion, and storage (Evans et al., 2019). Diverse systems for synchronized release of multiple drugs for cancer therapy have been designed based on single nanoparticles (Liao et al., 2014).

##### Heterostructured Nanoparticles

**FIGURE 2:**
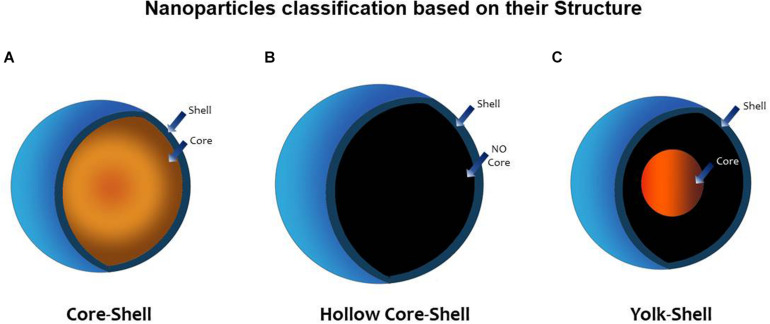
Nanoparticle classification based on its structure. **(A)** *Core–shell* (CS) nanoparticle (NP) possesses a central core (NP) surrounded by one or more layers of different material [shell(s)]. **(B)** *Hollow core–shell* structure or (*hollow* NP) refers to an NP without a core; the resulting empty space inside the shell can then be loaded with drugs, microRNA (miRNA), genes, peptides, and others that can now be released in a controlled manner. **(C)** Y*olk–shell* (YS) NP possesses a movable core that is located in a hollow cavity surrounded by a shell (Purbia and Paria, 2015).

In an attempt to increase the performance and functionality of nanomaterials, heterostructured nanoparticles composed of two or more different materials were created. This technology allowed for the design of advanced NPs with additional properties arising from the synergy of the different materials (Wei and Zhao, 2016). One method to concrete this effort was to coat nanoparticles with one or more layers. Nanoparticles created in this way can be classified as *core–shell* (CS), when a central core (NP) is surrounded by one or more layers of different material [shell(s)], or as *yolk–shell* (YS). when the a movable core is located in a hollow cavity surrounded by a shell (Purbia and Paria, 2015). A *hollow core–shell* structure or *hollow* NP is another term referred to a NP without a core; the resulting empty space inside the shell can then be loaded with drugs, microRNA (miRNA), genes, peptides, and others that can now be released in a controlled manner. *Janus nanoparticles* are a different type of nanomaterials; they possess a tunable asymmetric structure; their surface has two or more regions with different properties, which confer them unique properties as selective reactivity or directional interactions. The field of application is broad and innovative including its use as sensors, self-propelled carriers, or coatings (Agrawal and Agrawal, 2019; Figure 2).

### Nanoparticles as a Theragnostic Approach in Glioma

#### Nanoparticles as Radiosensitizers

Cancer tumors frequently contain a chemo and/or radioresistant subpopulation that survives and proliferates after standard treatments, contributing to the recurrence of a more aggressive tumor (Dahan et al., 2014; Yuan et al., 2018). Cancer stem cells (CSCs) represent this treatment-resistant subpopulation, and huge efforts are being focused on developing strategies to make them more amenable to current and novel therapies. The use of radiosensitizers is a potential approach to overcome radioresistance; however, its principal shortcoming is the lack of target specificity, which may lead to low concentrations in tumor tissue and toxic effects in healthy cells.

In this regard, nanoparticles have been tested as radiosensitizers agents and also as radiosensitizers carriers, showing promising results after photon and particle radiation (Caban-Toktas et al., 2020; Chung et al., 2020; Kazmi et al., 2020). For instance, Kunoh et al. developed DNA–gold nanoparticles complexes to work as radiosensitizers; they showed good cellular targeting and being effective in inducing cell death by mitotic catastrophe in glioma CSC after X-ray irradiation (Kunoh et al., 2019). Kefayat et al. also described good performance of folic-acid-coated gold nanocluster in radiosensitizing orthotopic C6 glioma tumor in a murine model (Kefayat et al., 2019). Folic acid receptors are differentially expressed in the luminal side of cancerous blood–brain barrier (BBB) endothelial cells as well as in cancer cells but not in normal tissues, which explain the higher concentration of these NP in glioma tumor when compared to a healthy brain tissue (Kefayat et al., 2019).

Furthermore, in order to target the glioma-resistant population specifically located in the tumor hypoxic niche, Hua et al. developed hypoxia-responsive yolk–shell nanoparticles (liposomes) by encapsulating radiosensitizer hydrophobic drugs [aniopep-2-poly-(metronidazoles)n and doxorubicin (DOX)] in hydrophilic polymers (PEG2000); these NPs were functionalized to target gliomas cells and release its content only under hypoxic conditions, increasing radiosensitization as shown *in vitro* and *in vivo* after systematic NP administration (Hua et al., 2018; Table 3).

#### Nanoparticles as Nanocarriers

The restricted permeability of the BBB has been one of the biggest challenges in the mission of effectively treating brain tumors. In nanomedicine, not all nanoparticles can efficiently cross this biological barrier despite their size and physicochemical characteristics; consequently, previously discussed strategies, such as *ligand targeting*, or improvements on the *enhanced permeability and retention (EPR) effect* are required.

While *ligand targeting* is an active targeting method and required NPs to be designed with this purpose in mind, *EPR effect* refers to a passive targeting mechanism common to all NPs. EPR effect relies on pathophysiological characteristics of tumor vs. healthy vessels as well as NP size, which is larger than individual conventional chemotherapeutics (usually < 1,000 Da). Due to their relatively larger size, NPs are not able to penetrate normal blood vessels but can easily cross diseased vessels such as those presented in brain tumors, leading to a selected distribution into cancer tissues. NP with diameters of at least 5–10 nm present reduced kidney excretion (by exceeding the clearance renal threshold of 40,000 Da), prolonged blood half-life, and better accumulation in the tissue of interest. For instance, the plasma half-life of doxorubicin increases from 5–10 min to 2–3 days when this is encapsulated into liposomes. In order to achieve better results from the application of organic nanoparticles such as liposomes, micelles, etc., some polymers such as PEG can be used to decrease NP aggregation and opsonization by plasma proteins, thus adding to the improved blood half-life (Hua et al., 2018).

Unfortunately, EPR effect is highly heterogeneous at inter- and intraindividual level, changing over time in the same tumor and even being dissimilar among different brain tumor lesions for the same patient. This, altogether, has led to clinical outcomes that does not match with preclinical results. In order to overcome these drawbacks, additional strategies to enhance BBB disruption and facilitate NP penetration have been applied. These strategies include pharmacological and physical methods such as sonoporation and radiation. Radiation can increase vascular permeability due to increased secretion of vascular endothelial growth factor (VEGF) and fibroblast growth factor (FGF) (Lee et al., 1995; Park et al., 2001). Thus, Lammers et al. (2007) showed a positive effect in the accumulation of DOX-loaded polymeric NP sized between 5 and 10 nm (31 and 65 kDa, respectively) when tumors were primed with different doses of radiation.

Overall, all the previously described strategies should be carefully weighted when trying to optimize the use of nanoparticles as nanocarriers. In this setting, when applied to an orthotopic glioblastoma model, the use of functionalized biodegradable polymeric nanoparticles coated or loaded with anticancer drug has been able to confer longer survivals in preclinical models (Yu et al., 2019). Among all the different nanocarriers (Figure 1), liposomes have been largely used. Preclinical studies using liposomes loaded with doxorubicin or coated with temozolomide showed higher concentrations of these drugs inside the brain when compared with the plasmatic levels; in these same models, survival benefit was also described (Zhao et al., 2018; Li et al., 2019). Noteworthy, liposomal doxorubicin has been clinically used in primary and recurrent high-grade glioma patients, and good biodistribution and decent outcomes were obtained; however, none of the studies were randomized controlled trials (RCTs) and were published just before or after the publication of the Stupp protocol (Fabel et al., 2001; Hau et al., 2004).

Overall, these results point the use of nanocarriers as a promising enhancer of effective therapies for the treatment of patients with glioma (Table 3).

#### Nanomachines

Nanomachines or nanobots are molecular self-propelled nanodevices considered as smart delivery systems that respond to specific triggers (Khawaja, 2011; Jager and Giacomelli, 2015; Saxena et al., 2015; Fu et al., 2017). DNA nanorobots are nanometric devices controlled by an aptamer-encoded logic gate, able to sense specific stimulus such as intracellular pH or cell surface ligands in order to activate and reconfigure its structure for delivery of different payloads. Li et al. reported on a DNA nanorobot created through the DNA origami method; this was programmed to unfold itself upon binding to caveolin molecules expressed in cancerous blood vessel endothelial cells in order to deliver thrombin into tumor vessels. The authors were able to prove this concept in a murine model of breast cancer, successfully inducing intratumorally vascular thrombosis that resulted in tumor necrosis and growth tumor inhibition (Li et al., 2018). This technology is revolutionizing the traditional way of treating different tumors and is a promising strategy to improve prognosis on brain tumor patients. Other novel approach introduced as a promising tool in the armamentarium for the treatment of glioma tumors is the use of stem cells. Along the next section, we will describe how the above-described nanotechnology has been coupled to engineer improved stem cell therapies for the treatment of brain cancer (Table 3).

## Applications of Nanoparticles in Stem Cell Glioma Therapy

Nanomedicine has extend the reach to several cancer treatment approaches such as radiotherapy, chemotherapy, immunotherapy, and others. In the case of stem cell therapies, improvements in several aspects are clearly needed. In an attempt to consolidate the translational potential of this approach, nanoparticles have been used to enhance safety and efficacy, stem cell tumor homing, and *in vivo* tracking after stem cell delivery. On the other hand, apart from nanoparticle surface modifications performed in an attempt to improve pharmacokinetics and pharmacodynamics parameters, stem cells appear as a reasonable option to overcome the suboptimal penetration, distribution, and retention associated to some nanomaterials when used as therapeutic nanocarriers. The use of stem cells in this context definitely add another option for a more targeted nanoparticle delivery. Thus, the benefit obtained from this combined approach using nanoparticles and stem cells is bidirectional.

### Nanoparticles for Stem Cells Genetic Engineering

Stem cells are known by their ability to serve as vehicles of antitumor cargoes. For this purpose, viral gene vectors have been traditionally used to transduce stem cells with a high degree of gene delivery efficiency resulting in constant payload production (anticancer proteins, cytokines, antibodies, viral vectors, etc.). Although newer generations of viral vectors present better safety profiles, these vectors have been associated with immunotoxicity as a response to viral proteins production or potential viral replication. They also would carry the hypothetical risk of uncontrolled viral genome integration and insertional mutagenesis, latent virus activation, and inflammatory responses leading to demyelination or neurodegeneration (Dewey et al., 1999; Mangraviti et al., 2016). In this setting, nanoparticle-based gene delivery represents an attractive non-viral strategy to bioengineer stem cells. Different from commercially available reagents such as Lipofectamine 2000 (Thermo Fisher Scientific, Waltham, MA) nanoparticles may represent a less toxic and more effective approach for gene delivery.

Our group reported on the use of biodegradable polymeric nanoparticles based on poly(beta-amino ester)s (PBAEs) to enable effective BMP4 gene delivery on human A-MSC, allowing for higher transfection rates than those of commercially available reagents. Transfected MSC retained their multipotency and their tumor-homing capacity and were functional, leading to extended survival in a rat orthotopic GBM model (Yong et al., 2009). Huang et al. also reported on the use of nanoparticles for stem cell bioengineering; using hyaluronic acid (HA)-decorated superparamagnetic iron oxide nanoparticles as part of a magnetic ternary nanohybrid (MTN), the group was able to construct tumor necrosis factor-related apoptosis-inducing ligand (TRAIL)-secreting human mesenchymal stromal cell (hMSC). Decoration with CD44-binding HA and magnetic forces were used in this approach to increase cellular uptake of MTN. Impairment in tumor-homing properties were not observed (Huang R. Y. et al., 2019). Overall, nanoparticles raise as an option of safe and efficient gene delivery for stem cell; thus, helping stem cell therapy to achieve its maximal therapeutic potential.

### Nanoparticles as Stem Cells Payloads

In the treatment of several malignancies, different nanoplatforms acting themselves as anticancer agents (Mooney et al., 2014a) or as carriers for these anticancer drugs (Mooney et al., 2014b) have been delivered locally and systemically. Recent advances in nanomedicine have allowed tuning nanoparticles properties in such a way that crossing the BBB and reaching brain tumors is now possible. However, there is a fine line between three factors: (1) the ideal size that a nanoparticle must have to easily cross the BBB (up to150 nm, optimal passage if < 15 nm) (Gao and Jiang, 2006), (2) being big enough to still be able to carry enough payload, and (3) being small enough to avoid engulfment by the mononuclear phagocyte system but still contain all the necessary ligands to assure specific cancer targeting (Owens and Peppas, 2006).

Furthermore, even if researchers could secure that nanoparticles reach the glioma tumor bulk, there exist other potential drawbacks that are imperative to highlight; they are related to the presence and location of glioma cancer stem cells (1) Nanoparticles are neither able to track infiltrative glioma cells leaving the tumor bulk to colonize distal healthy brain parenchyma nor (2) to reach the necrotic glioma core where blood flow is impaired. These areas do not present an EPR effect, which would facilitate nanoparticles to distribute across other areas of the tumor. Allowing nanoparticles to access the hypoxic central core would be crucial, as the treatment-resistant subpopulation of glioma cancer cells would predominantly locate in that area (Table 4). Even after active targeting strategies including ligand targeting and microenvironment-related targeting (delivering payload depending on pH, temperature, etc.) (Koo et al., 2006; Bernardi et al., 2008; Madhankumar et al., 2009; Hadjipanayis et al., 2010; Wang et al., 2011), nanoparticles alone are still insufficient and would be unlikely to overcome the above-mentioned roadblocks.

In this scenario, coupling stem cell therapy to nanotherapeutics offers the possibility to solve the previously stated dilemma regarding the inadequate distribution of therapeutic nanoparticles to the hypoxic glioma core and distant infiltrative tumor foci. Thus, stem cells could extend the reach for nanoparticles to penetrate these areas. This approach implies nanoparticles to be conjugated to stem cell surfaces or internalized before migrating toward malignant gliomas. Furthermore, the internalization of nanoparticles inside stem cells would allow them to be up to fivefold larger than the usual nanoparticles used in cancer therapy, without entailing problems in crossing the BBB or a higher risk to be engulfed by macrophages or lymphocytes (Koo et al., 2006; Bernardi et al., 2008; Madhankumar et al., 2009; Hadjipanayis et al., 2010; Wang et al., 2011). This increase in the nanoparticles’ longitudinal size translates into an approximately 125-fold increase in the nanoparticle load potential (by a volume-based, three-dimensional factor of 5) (Koo et al., 2006; Bernardi et al., 2008; Madhankumar et al., 2009; Hadjipanayis et al., 2010; Wang et al., 2011).

In this same line, the Aboody group demonstrated that *neural stem cells* were able to improve intracranial nanoparticle retention and tumor-selective distribution in an *in vivo* model by coupling huge nanoparticles to NSC surface (Mooney et al., 2014b). Taking advantage from the significant differences in the environmental pH between tumor and healthy tissues, the Aboody group loaded FDA-approved NSC cell with pH−sensitive doxorubicin−loaded mesoporous silica nanoparticles (MSN−Dox); the authors were able to tune nanoparticles properties to delay doxorubicin toxicity, allowing NSC to home into glioma tumors and deliver its payload only after arriving at the acidic tumor microenvironment. The approach led to a significant difference in survival when studied in a preclinical *in vivo* model (Cheng Y. et al., 2013). The same group also evaluated the role of NSC loaded with gold nanorods (AuNRs) to improve plasmonic photothermal therapy (aka thermal ablation), where the nanoparticles help to convert light into heat, aiming to eliminate cancerous tumor cells. The authors found that intratumor injections of AuNR-loaded NSC improved AuNRs distribution inside the tumor bulk when compared to locally injected free AuNRs in a brain metastasis heterotopic *in vivo* model (Mooney et al., 2014a).

The role of *mesenchymal stem cells* as nanoparticle carriers has also been investigated. Polymeric nanoparticles (paclitaxel-encapsulated PLGA nanoparticles) were loaded into BM-MSC. Osteogenesis, adipogenesis (chondrogenesis was not evaluated), and tumor homing were not affected by nanoparticle inclusion. The approach was associated with improved survival in a rat orthotopic glioma model when the modified MSCs were injected in the contralateral hemisphere (Wang et al., 2018). A similar approach to the one described by the Aboody group was performed on modified MSCs by loading them with gold nanoparticles (nanostarts) to improve phototherapy. Although studied in a heterotopic model of prostate cancer, the results support the use of MSC to maximize clinically relevant gold nanoparticles’ optical–electronic properties by increasing nanoparticle intratumor distribution (Huang L. et al., 2019).

### Nanoparticles to Modulate Stem Cell Tumor Homing

Several *tumor cytokines* and *stem cells surface proteins* have been involved in enhancing MSC migration toward glioma tumors; however, no specific mechanism has been described yet. *Tumor cytokines* such as endothelial cell growth factor (EGF), platelet-derived growth factor (PDGF), VEGF, tumor growth factor β1 (TGF-β1), interleukin 8 (IL-8), monocyte chemoattractant protein-1 (MCP-1), and stromal cell-derived factor 1 alpha (SDF-1α) as well as *stem cells surface proteins* such as CD44, CXC chemokine receptor 4 (CXCR-4), integrin α4, and TGF-β receptors have been associated with increased MSC homing in gliomas (Young et al., 2014; Yamazoe et al., 2015).

The impact on stem cell behavior after being loaded with nanoparticles for different purposes has not been the principal focus of research. However, there already exist reports describing the increase in migration toward cancer cells after loading hMSC with iron oxide nanoparticles. This would be related to the overexpression of EGFR observed after nanoparticle inclusion and the characteristic elevated production of EGF by colon cancer cells used in the *in vitro* Boyden chamber experiments (Chung et al., 2011). Interestingly, the same trend has been observed when human BM-MSC were labeled with ferucarbotran nanoparticles and protamine. Using cellular magnetic resonance imagining (MRI) to track the labeled stem cells, increased BM-MSC migration toward *in vitro* and *in vivo* glioma models was found, and the SDF-1/CXCR4 signaling axis was associated to this phenomenon (Chien et al., 2011).

### Nanoparticles for Tracking Stem Cells During Glioma Therapy

Stem-cell-based therapies rely on the ability of the grafted cells to target the organ of interest. In case of malignant gliomas, it is crucial to ensure stem cell tumor homing. In the preclinical setting, conventional methods for tracking migration and final fade of stem cells are traditionally based on bioluminescence imaging; however, poor spatial distribution and lack of translational applicability made necessary to establish a reasonably translational method that can be easily applied in a clinical setup.

Cellular MRI-based tracking technologies have risen as gold standard for non-invasive, real-time monitoring of transplanted stem cells (Kim et al., 2011). This approach would allow the study of stem cell biodistribution, migration, survival, and even differentiation with high spatial resolution and without the need for ionizing radiation. To make this possible, stem cells will require being labeled with magnetic nanoparticles. Although several options exit, magnetic iron oxide nanoparticles such superparamagnetic iron oxide nanoparticles (SPIONs) have been commonly used for this purpose (Cromer Berman et al., 2011).

The conjugation of stem cells and SPIONs has allowed for tracking MSC migration and homing into glioma tumors in a rodent glioma model without compromising such migratory capacity (Wu et al., 2008; Menon et al., 2012). NSCs have also been widely studied in this regard (Spina et al., 1975; Neri et al., 2008). After 1 month of follow-up, it was demonstrated that SPIONs would not impair multipotency, cell survival, or proliferation (Agha-Hosseini et al., 2010). Furthermore, a NSC migration speed of 50–70 μm/day has been calculated after the cells were loaded with ferumoxide (SPION + dextran) (Flexman et al., 2011). Clinically relevant results were those presented by Thu et al. The group showed that loading FDA-approved NSC with ferumoxide–protamine complex nanoparticles did not impaired humor-homing properties in a murine glioma model (Thu et al., 2009; Auffinger et al., 2013). Gutova et al. also reported on similar findings when using ultrasmall superparamagnetic iron oxide nanoparticles (USPIONs) in clinically graded nanoparticles and FDA-approved NSC (Gutova et al., 2013). Currently, different complementary imaging modalities and nanoparticles stem-cell coupling techniques are being studied (Egawa et al., 2015; Cheng S. H. et al., 2016; Qiao et al., 2018).

Even when this approach was first evaluated in the clinical setting around 2006 (Zhu et al., 2006) and has been used in different pathologies and other cancers (de Vries et al., 2005), glioma patients have not yet harnessed the benefit of the clinical applicability of this technology. This could be related to the difficulties in obtaining long-term follow-up of nanoparticle-labeled stem cells, as their self-renewal capacity render less nanoparticle concentration through each replicative cell cycle.

## Challenge, Potential Pitfalls, and Future Perspective

Challenge and pitfalls associated with this relatively novel approach is proper of any disruptive technology. The ethical concerns associated with the use of particular stem cells, while seemingly addressed with modern techniques, need to be further discussed before extensive use can be assumed (Ramos-Zúñiga et al., 2012). Clinical endeavors utilizing stem cells as potential therapeutic tools in glioma patients have already glimpsed relative success. In this setting, careful and individualized selection of specific types of stem cell will be key in in future clinical applications for these patients. For instance, we concentrate our efforts in the application of adipose-derived MSCs, which can be easily obtained from the same patient. Although still in preclinical phase, we expect them to be rapidly bioengineered and used for autologous transplantation, thus allowing for an individualized and expedited process so the patients can therapeutically receive them even at time of surgery.

The introduction of the nanotechnology in stem cell therapies has shown to be beneficial and hopefully will keep turning stem cell therapies into a less worrisome and more controlled therapeutic strategy. To date, we have explored NP for stem cell bioengineering and cell tracking; however, we believe that their malleability allows for further uses such as the ones previously described, alone or in combination, and even for stem cell functionalization (Kim et al., 2011; Bishop et al., 2016; Mangraviti et al., 2016; Wilson et al., 2017a, b; Tian et al., 2020).

Finally, the combination of these therapies should not be limited to only nanoparticles and stem cells; this combined approach will need to explore if further value can be obtained by coupling with additional fields such as radiotherapy, thermotherapy, targeted systemic therapies, focused ultrasound, and other novel diagnostic techniques such as ultrahigh magnetic strength imaging and novel radiotracers in order to maximize its benefits.

## Author Contributions

HR-G and KA-E screened titles for relevance and abstracted the data from the eligible full text articles. SK, AQ-H, and DT critically revised the manuscript with input from the entire team. HR-G and KA-E created the figures and tables. HR-G, KA-E, and DT worked on study conception and design. All authors analyzed and interpreted the data, drafted the manuscript, and read and approved the final draft.

## Conflict of Interest

The authors declare that the research was conducted in the absence of any commercial or financial relationships that could be construed as a potential conflict of interest.
